# Bis[hexa­amminecobalt(III)] penta­chloride nitrate

**DOI:** 10.1107/S1600536812021332

**Published:** 2012-05-16

**Authors:** Qihui Wu, Chunyu Du, Yang Lv, Guoliang Chen, Qinhe Pan

**Affiliations:** a Department of Materials and Chemical Engineering, Ministry of Education Key Laboratory of Advanced Materials of Tropical Island Resources, Hainan University, Haikou 570228, People’s Republic of China

## Abstract

The title compound, [Co(NH_3_)_6_]_2_Cl_5_(NO_3_), was obtained under hydro­thermal conditions. The asymmetric unit contains three Co^3+^ ions, one lying on an inversion center and the other two located at 2/*m* positions. All Co^3+^ ions are six-coordinated by NH_3_ mol­ecules, forming [Co(NH_3_)_6_]^3+^ octahedra, with Co—N distances in the range 1.945 (4)–1.967 (3) Å. The nitrate N atom and one of the O atoms lie at a mirror plane. Among the Cl^−^ anions, one lies in a general position, one on a twofold axis and two on a mirror plane. N—H⋯O and N—H⋯Cl hydrogen bonds link the cations and anions into a three-dimensional network.

## Related literature

For metal phosphates and germanates prepared using metal complexes as templates, see: Wang *et al.* (2003**a* ▶,b* ▶); Pan *et al.* (2005 ▶, 2008 ▶). For our continued research inter­est focused on the synthesis of microporous open-framework metal-organic hybride materials by introducing transition metal complexes as templates, see: Pan *et al.* (2010**a* ▶,b* ▶, 2011 ▶); Tong & Pan (2011 ▶); Liang *et al.* (2011 ▶). For a structure containing a [Co(NH_3_)_6_]^3+^ cation, see: Han *et al.* (2012 ▶).
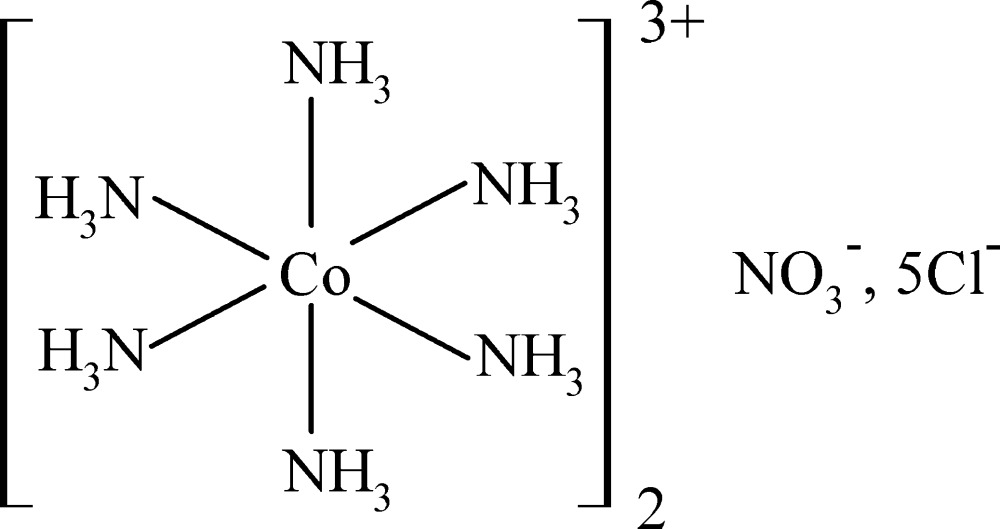


## Experimental

### 

#### Crystal data

[Co(NH_3_)_6_]_2_Cl_5_(NO_3_)
*M* *_r_* = 561.53
Monoclinic, 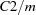

*a* = 21.118 (4) Å
*b* = 14.985 (3) Å
*c* = 6.8491 (11) Å
β = 92.147 (3)°
*V* = 2165.8 (6) Å^3^
*Z* = 4
Mo *K*α radiation
μ = 2.18 mm^−1^
*T* = 296 K
0.20 × 0.12 × 0.10 mm

#### Data collection

Bruker APEXII CCD area-detector diffractometer
Absorption correction: multi-scan (*SADABS*; Bruker, 2005 ▶) *T*_min_ = 0.738, *T*_max_ = 0.770
7927 measured reflections
2813 independent reflections
1870 reflections with *I* > 2σ(*I*)
*R*_int_ = 0.046

#### Refinement

*R*[*F* ^2^ > 2σ(*F* ^2^)] = 0.050
*wR*(*F* ^2^) = 0.140
*S* = 1.02
2813 reflections
119 parameters
H-atom parameters constrained
Δρ_max_ = 0.65 e Å^−3^
Δρ_min_ = −0.71 e Å^−3^

### 

Data collection: *APEX2* (Bruker, 2005 ▶); cell refinement: *SAINT* (Bruker, 2005 ▶); data reduction: *SAINT*; program(s) used to solve structure: *SHELXS97* (Sheldrick, 2008 ▶); program(s) used to refine structure: *SHELXL97* (Sheldrick, 2008 ▶); molecular graphics: *SHELXTL* (Sheldrick, 2008 ▶); software used to prepare material for publication: *SHELXTL*.

## Supplementary Material

Crystal structure: contains datablock(s) I, global. DOI: 10.1107/S1600536812021332/yk2056sup1.cif

Structure factors: contains datablock(s) I. DOI: 10.1107/S1600536812021332/yk2056Isup2.hkl

Additional supplementary materials:  crystallographic information; 3D view; checkCIF report

## Figures and Tables

**Table 1:** Hydrogen-bond geometry (Å, °)

*D*—H⋯*A*	*D*—H	H⋯*A*	*D*⋯*A*	*D*—H⋯*A*
N1—H1*A*⋯Cl1	0.89	2.89	3.427 (4)	120
N1—H1*A*⋯Cl3^i^	0.89	2.90	3.484 (4)	125
N1—H1*B*⋯Cl1^ii^	0.89	2.59	3.410 (4)	154
N1—H1*C*⋯Cl3^iii^	0.89	2.63	3.431 (4)	150
N3—H3*A*⋯O2^iv^	0.89	2.53	3.155 (6)	128
N4—H4*A*⋯Cl3^v^	0.89	2.79	3.287 (4)	117
N4—H4*B*⋯Cl4	0.89	2.62	3.448 (5)	155
N4—H4*C*⋯Cl2^v^	0.89	2.73	3.321 (4)	125
N5—H5*A*⋯Cl1^vi^	0.89	2.77	3.375 (4)	127
N5—H5*A*⋯Cl4^vii^	0.89	2.91	3.456 (4)	122
N5—H5*B*⋯O2^vii^	0.89	2.17	3.048 (5)	168
N5—H5*C*⋯Cl3^vi^	0.89	2.56	3.337 (4)	146
N6—H6*A*⋯Cl4^vii^	0.89	2.76	3.339 (4)	124
N6—H6*C*⋯Cl3^viii^	0.89	2.93	3.445 (4)	118
N7—H7*A*⋯Cl4^iv^	0.89	2.78	3.368 (4)	125
N7—H7*B*⋯Cl3^viii^	0.89	2.87	3.394 (4)	119

Symmetry codes: (i) 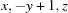; (ii) 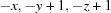; (iii) 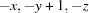; (iv) 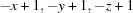; (v) 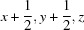; (vi) 
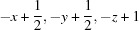
; (vii) 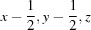; (viii) 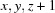.

**Fig. 1.**
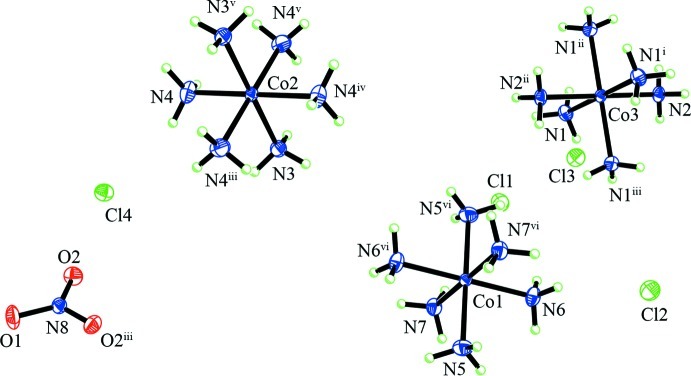
A view of the asymmetric unit of title compound showing the atom labelling scheme. Ellipsoids are drawn at the 30% probability level. [Symmetry codes: (i) -x, 1 - y, -z; (ii) -x, y, -z; (iii) x, 1 - y, z; (iv) 1 - x, 1 - y, -z; (v) 1 - x, y, -z; (vi) 1/2 - x, 1/2 - y, 1 - z]. (iv) x, -y + 1, z; (v) -x, -y + 1, -z; (vi) -x, y, -z.

## supplementary crystallographic information

### Comment

Rencently, more attention has been paid to transition metal complexes, because they can to be employed as templates in the synthesis of various open-framework materials, including metal phosphates (Wang *et al.*, 2003**a*,b*) and germanates (Pan *et al.*, 2005,2008). Our continued interest has been focused on the synthesis of microporous open-framework metal-organic hybride materials by introducing transition metal complexes as templates (Pan *et al.*, 2010**a*,b*, 2011; Tong & Pan, 2011; Liang *et al.*, 2011). Unexpectedly, the title compound, [Co(NH_3_)_6_]_2_(NO_3_)Cl_5_, was obtained.

The title compound is composed of [Co(NH_3_)_6_]^3+^ cations and the counterions Cl^-^ and NO_3_^-^, as shown in Fig. 1. The asymmetric part of this crystal structure contains three Co^3+^ ions; one is located on an inversion center, and the other two are positioned on the twofold rotation axis with center of symmetry (2/m). All Co(III) ions are six coordinated by NH_3_ molecules to form [Co(NH_3_)_6_]^3+^ cations, having a slightly distorted octahedral geometry, as in the structure of [Co(NH_3_)_6_]_3_[Zn_8_(HPO_4_)_8_(PO_4_)_2_](PO_4_) (Han *et al.*, 2012). The Co—N bond distances are in the range from 1.945 (4) to 1.967 (3) Å. For the counterions, the N8 atom of NO_3_^-^ anion is located on mirror plane and displays a trigonal geometry by bonded to three O atoms with the N—O distances of 1.234 (6)–1.253 (4) Å. The Cl^-^ anions are located in different positions: inversion center for Cl2, mirror plane for Cl1 and Cl4, and general position for Cl3. The [Co(NH_3_)_6_]^3+^ cations interact with the counterions Cl^-^ and NO_3_^-^*via* hydrogen bonds; the distances of N—H···O hydrogen bonds are in the range 3.048 (5)–3.155 (6) Å, and the distances of N—H···Cl hydrogen bonds lie in the range from 3.287 (4) to 3.484 (4) Å (Table 1), to form an extensive three-dimensional hydrogen-bonding network.

### Experimental

In a typical synthesis, a mixture of Cd(NO_3_)_2_.4H_2_O (0.231 g), pyromellitic acid (0.0254 g), [Co(NH_3_)_6_]Cl_3_ (0.03 g), NaOH (0.016 g) and H_2_O (10 ml) were added in a 20 ml Teflon-lined reactor under autogenous pressure at 100°C for 3 days. Yellow rod-like crystals were obtained.

### Refinement

All H atoms were positioned geometrically (N—H = 0.89 Å) and allowed to ride on their parent atoms, with *U*_iso_(H) = 1.2*U*_eq_(parent atom).

### Crystal data

**Table:**

[Co(NH_3_)_6_]_2_Cl_5_(NO_3_)	*F*(000) = 1160
*M**_r_* = 561.53	*D*_x_ = 1.722 Mg m^−^^3^
Monoclinic, *C*2/*m*	Mo *K*α radiation, λ = 0.71073 Å
Hall symbol: -C 2y	Cell parameters from 7927 reflections
*a* = 21.118 (4) Å	θ = 1.7–28.4°
*b* = 14.985 (3) Å	µ = 2.18 mm^−^^1^
*c* = 6.8491 (11) Å	*T* = 296 K
β = 92.147 (3)°	Rod, yellow
*V* = 2165.8 (6) Å^3^	0.2 × 0.12 × 0.10 mm
*Z* = 4	

### Data collection

**Table:**

Bruker APEXII CCD area-detector diffractometer	2813 independent reflections
Radiation source: fine-focus sealed tube	1870 reflections with *I* > 2σ(*I*)
Graphite monochromator	*R*_int_ = 0.046
Detector resolution: 83.66 pixels mm^-1^	θ_max_ = 28.4°, θ_min_ = 1.7°
φ and ω scans	*h* = −27→28
Absorption correction: multi-scan (*SADABS*; Bruker, 2005)	*k* = −19→20
*T*_min_ = 0.738, *T*_max_ = 0.770	*l* = −6→9
7927 measured reflections	

### Refinement

**Table:**

Refinement on *F*^2^	Primary atom site location: structure-invariant direct methods
Least-squares matrix: full	Secondary atom site location: difference Fourier map
*R*[*F*^2^ > 2σ(*F*^2^)] = 0.050	Hydrogen site location: inferred from neighbouring sites
*wR*(*F*^2^) = 0.140	H-atom parameters constrained
*S* = 1.02	*w* = 1/[σ^2^(*F*_o_^2^) + (0.0641*P*)^2^ + 4.8344*P*] where *P* = (*F*_o_^2^ + 2*F*_c_^2^)/3
2813 reflections	(Δ/σ)_max_ < 0.001
119 parameters	Δρ_max_ = 0.65 e Å^−^^3^
0 restraints	Δρ_min_ = −0.71 e Å^−^^3^

### Special details

**Table:**

Geometry. All s.u.'s (except the s.u. in the dihedral angle between two l.s. planes) are estimated using the full covariance matrix. The cell s.u.'s are taken into account individually in the estimation of s.u.'s in distances, angles and torsion angles; correlations between s.u.'s in cell parameters are only used when they are defined by crystal symmetry. An approximate (isotropic) treatment of cell s.u.'s is used for estimating s.u.'s involving l.s. planes.
Refinement. Refinement of *F*^2^ against ALL reflections. The weighted *R*-factor *wR* and goodness of fit *S* are based on *F*^2^, conventional *R*-factors *R* are based on *F*, with *F* set to zero for negative *F*^2^. The threshold expression of *F*^2^ > σ(*F*^2^) is used only for calculating *R*-factors(gt) *etc*. and is not relevant to the choice of reflections for refinement. *R*-factors based on *F*^2^ are statistically about twice as large as those based on *F*, and *R*- factors based on ALL data will be even larger.

### Fractional atomic coordinates and isotropic or equivalent isotropic displacement parameters (Å^2^)

**Table:**

	*x*	*y*	*z*	*U*_iso_*/*U*_eq_	
Co1	0.2500	0.2500	0.5000	0.0193 (2)	
N7	0.25395 (17)	0.3197 (3)	0.7422 (5)	0.0329 (9)	
H7A	0.2923	0.3440	0.7585	0.040*	
H7B	0.2465	0.2842	0.8429	0.040*	
H7C	0.2249	0.3627	0.7353	0.040*	
N6	0.16170 (16)	0.2158 (3)	0.5415 (5)	0.0342 (9)	
H6A	0.1477	0.1812	0.4434	0.041*	
H6B	0.1378	0.2646	0.5463	0.041*	
H6C	0.1596	0.1861	0.6535	0.041*	
N5	0.27844 (19)	0.1439 (3)	0.6414 (6)	0.0401 (10)	
H5A	0.2811	0.0984	0.5585	0.048*	
H5B	0.2509	0.1306	0.7323	0.048*	
H5C	0.3164	0.1541	0.6982	0.048*	
Co2	0.5000	0.5000	0.0000	0.0212 (3)	
Cl4	0.66930 (8)	0.5000	0.4229 (2)	0.0350 (4)	
N4	0.55640 (18)	0.5926 (3)	0.1076 (6)	0.0411 (10)	
H4A	0.5852	0.6062	0.0208	0.049*	
H4B	0.5758	0.5727	0.2168	0.049*	
H4C	0.5339	0.6410	0.1341	0.049*	
N3	0.4522 (2)	0.5000	0.2394 (7)	0.0329 (12)	
H3A	0.4278	0.4517	0.2435	0.040*	
H3B	0.4787	0.5000	0.3435	0.040*	
Co3	0.0000	0.5000	0.0000	0.0208 (3)	
N2	−0.0927 (2)	0.5000	−0.0085 (7)	0.0289 (11)	
H2A	−0.1072	0.4517	−0.0712	0.035*	
H2B	−0.1075	0.5000	0.1114	0.035*	
N1	0.00050 (17)	0.5934 (3)	0.2019 (5)	0.0330 (9)	
H1A	0.0403	0.6075	0.2360	0.040*	
H1B	−0.0191	0.5734	0.3061	0.040*	
H1C	−0.0195	0.6415	0.1551	0.040*	
Cl3	0.12244 (5)	0.28432 (8)	0.00154 (16)	0.0351 (3)	
Cl2	0.0000	0.19040 (14)	0.5000	0.0485 (5)	
Cl1	0.11173 (8)	0.5000	0.5167 (2)	0.0459 (5)	
O2	0.68054 (15)	0.5722 (2)	0.9173 (5)	0.0392 (8)	
N8	0.7099 (2)	0.5000	0.9396 (7)	0.0291 (11)	
O1	0.7671 (2)	0.5000	0.9832 (8)	0.0507 (14)	

### Atomic displacement parameters (Å^2^)

**Table:**

	*U*^11^	*U*^22^	*U*^33^	*U*^12^	*U*^13^	*U*^23^
Co1	0.0190 (4)	0.0185 (4)	0.0206 (4)	0.0001 (3)	0.0021 (3)	0.0002 (3)
N7	0.032 (2)	0.039 (2)	0.028 (2)	−0.0044 (17)	0.0031 (15)	−0.0072 (15)
N6	0.0265 (19)	0.036 (2)	0.041 (2)	−0.0052 (17)	0.0084 (16)	−0.0079 (18)
N5	0.046 (2)	0.032 (2)	0.042 (2)	0.0034 (19)	−0.0037 (18)	0.0093 (17)
Co2	0.0179 (5)	0.0211 (6)	0.0247 (6)	0.000	0.0025 (4)	0.000
Cl4	0.0419 (9)	0.0284 (8)	0.0347 (9)	0.000	−0.0006 (7)	0.000
N4	0.033 (2)	0.044 (3)	0.047 (2)	−0.0102 (19)	0.0093 (18)	−0.0124 (19)
N3	0.027 (3)	0.043 (3)	0.028 (3)	0.000	0.005 (2)	0.000
Co3	0.0165 (5)	0.0220 (6)	0.0239 (6)	0.000	0.0019 (4)	0.000
N2	0.017 (2)	0.029 (3)	0.040 (3)	0.000	−0.001 (2)	0.000
N1	0.030 (2)	0.035 (2)	0.034 (2)	0.0027 (17)	−0.0011 (16)	−0.0044 (16)
Cl3	0.0334 (6)	0.0315 (6)	0.0405 (7)	0.0048 (5)	0.0037 (5)	0.0012 (5)
Cl2	0.0462 (10)	0.0521 (12)	0.0478 (11)	0.000	0.0079 (8)	0.000
Cl1	0.0361 (9)	0.0683 (13)	0.0337 (9)	0.000	0.0056 (7)	0.000
O2	0.0395 (19)	0.036 (2)	0.043 (2)	0.0092 (15)	0.0046 (15)	−0.0019 (14)
N8	0.024 (3)	0.037 (3)	0.027 (3)	0.000	0.004 (2)	0.000
O1	0.022 (2)	0.053 (4)	0.077 (4)	0.000	−0.005 (2)	0.000

### Geometric parameters (Å, º)

**Table:**

Co1—N5^i^	1.945 (4)	Co2—N3^ii^	1.958 (5)
Co1—N5	1.945 (4)	N4—H4A	0.8900
Co1—N7	1.960 (3)	N4—H4B	0.8900
Co1—N7^i^	1.960 (3)	N4—H4C	0.8900
Co1—N6	1.965 (3)	N3—H3A	0.8900
Co1—N6^i^	1.965 (3)	N3—H3B	0.8900
N7—H7A	0.8900	Co3—N2	1.956 (5)
N7—H7B	0.8900	Co3—N2^v^	1.956 (5)
N7—H7C	0.8900	Co3—N1	1.967 (3)
N6—H6A	0.8900	Co3—N1^vi^	1.967 (3)
N6—H6B	0.8900	Co3—N1^v^	1.967 (3)
N6—H6C	0.8900	Co3—N1^iv^	1.967 (3)
N5—H5A	0.8900	N2—H2A	0.8900
N5—H5B	0.8900	N2—H2B	0.8900
N5—H5C	0.8900	N1—H1A	0.8900
Co2—N4	1.955 (4)	N1—H1B	0.8900
Co2—N4^ii^	1.955 (4)	N1—H1C	0.8900
Co2—N4^iii^	1.955 (4)	O2—N8	1.253 (4)
Co2—N4^iv^	1.955 (4)	N8—O1	1.234 (6)
Co2—N3	1.958 (5)	N8—O2^iv^	1.253 (4)
			
N5^i^—Co1—N5	180.000 (1)	N4^iv^—Co2—N3	90.59 (16)
N5^i^—Co1—N7	89.33 (16)	N4—Co2—N3^ii^	89.41 (16)
N5—Co1—N7	90.67 (16)	N4^ii^—Co2—N3^ii^	90.59 (16)
N5^i^—Co1—N7^i^	90.67 (16)	N4^iii^—Co2—N3^ii^	90.59 (16)
N5—Co1—N7^i^	89.33 (16)	N4^iv^—Co2—N3^ii^	89.41 (16)
N7—Co1—N7^i^	180.0	N3—Co2—N3^ii^	180.000 (1)
N5^i^—Co1—N6	90.47 (17)	Co2—N4—H4A	109.5
N5—Co1—N6	89.53 (17)	Co2—N4—H4B	109.5
N7—Co1—N6	91.56 (15)	H4A—N4—H4B	109.5
N7^i^—Co1—N6	88.44 (15)	Co2—N4—H4C	109.5
N5^i^—Co1—N6^i^	89.53 (17)	H4A—N4—H4C	109.5
N5—Co1—N6^i^	90.47 (17)	H4B—N4—H4C	109.5
N7—Co1—N6^i^	88.44 (15)	Co2—N3—H3A	110.1
N7^i^—Co1—N6^i^	91.56 (15)	Co2—N3—H3B	110.0
N6—Co1—N6^i^	180.00 (6)	H3A—N3—H3B	108.9
Co1—N7—H7A	109.5	N2—Co3—N2^v^	180.0
Co1—N7—H7B	109.5	N2—Co3—N1	90.00 (15)
H7A—N7—H7B	109.5	N2^v^—Co3—N1	90.00 (15)
Co1—N7—H7C	109.5	N2—Co3—N1^vi^	90.00 (15)
H7A—N7—H7C	109.5	N2^v^—Co3—N1^vi^	90.00 (15)
H7B—N7—H7C	109.5	N1—Co3—N1^vi^	89.3 (2)
Co1—N6—H6A	109.5	N2—Co3—N1^v^	90.00 (15)
Co1—N6—H6B	109.5	N2^v^—Co3—N1^v^	90.00 (15)
H6A—N6—H6B	109.5	N1—Co3—N1^v^	180.0 (2)
Co1—N6—H6C	109.5	N1^vi^—Co3—N1^v^	90.7 (2)
H6A—N6—H6C	109.5	N2—Co3—N1^iv^	90.00 (15)
H6B—N6—H6C	109.5	N2^v^—Co3—N1^iv^	90.00 (15)
Co1—N5—H5A	109.5	N1—Co3—N1^iv^	90.7 (2)
Co1—N5—H5B	109.5	N1^vi^—Co3—N1^iv^	180.00 (16)
H5A—N5—H5B	109.5	N1^v^—Co3—N1^iv^	89.3 (2)
Co1—N5—H5C	109.5	Co3—N2—H2A	109.9
H5A—N5—H5C	109.5	Co3—N2—H2B	111.0
H5B—N5—H5C	109.5	H2A—N2—H2B	108.6
N4—Co2—N4^ii^	180.0	Co3—N1—H1A	109.5
N4—Co2—N4^iii^	89.6 (3)	Co3—N1—H1B	109.5
N4^ii^—Co2—N4^iii^	90.4 (3)	H1A—N1—H1B	109.5
N4—Co2—N4^iv^	90.4 (3)	Co3—N1—H1C	109.5
N4^ii^—Co2—N4^iv^	89.6 (3)	H1A—N1—H1C	109.5
N4^iii^—Co2—N4^iv^	180.00 (17)	H1B—N1—H1C	109.5
N4—Co2—N3	90.59 (16)	O1—N8—O2^iv^	120.3 (3)
N4^ii^—Co2—N3	89.41 (16)	O1—N8—O2	120.3 (3)
N4^iii^—Co2—N3	89.41 (16)	O2^iv^—N8—O2	119.4 (5)

Symmetry codes: (i) −*x*+1/2, −*y*+1/2, −*z*+1; (ii) −*x*+1, −*y*+1, −*z*; (iii) −*x*+1, *y*, −*z*; (iv) *x*, −*y*+1, *z*; (v) −*x*, −*y*+1, −*z*; (vi) −*x*, *y*, −*z*.

### Hydrogen-bond geometry (Å, º)

**Table:**

*D*—H···*A*	*D*—H	H···*A*	*D*···*A*	*D*—H···*A*
N1—H1*A*···Cl1	0.89	2.89	3.427 (4)	120
N1—H1*A*···Cl3^iv^	0.89	2.90	3.484 (4)	125
N1—H1*B*···Cl1^vii^	0.89	2.59	3.410 (4)	154
N1—H1*C*···Cl3^v^	0.89	2.63	3.431 (4)	150
N3—H3*A*···O2^viii^	0.89	2.53	3.155 (6)	128
N4—H4*A*···Cl3^ix^	0.89	2.79	3.287 (4)	117
N4—H4*B*···Cl4	0.89	2.62	3.448 (5)	155
N4—H4*C*···Cl2^ix^	0.89	2.73	3.321 (4)	125
N5—H5*A*···Cl1^i^	0.89	2.77	3.375 (4)	127
N5—H5*A*···Cl4^x^	0.89	2.91	3.456 (4)	122
N5—H5*B*···O2^x^	0.89	2.17	3.048 (5)	168
N5—H5*C*···Cl3^i^	0.89	2.56	3.337 (4)	146
N6—H6*A*···Cl4^x^	0.89	2.76	3.339 (4)	124
N6—H6*C*···Cl3^xi^	0.89	2.93	3.445 (4)	118
N7—H7*A*···Cl4^viii^	0.89	2.78	3.368 (4)	125
N7—H7*B*···Cl3^xi^	0.89	2.87	3.394 (4)	119

Symmetry codes: (i) −*x*+1/2, −*y*+1/2, −*z*+1; (iv) *x*, −*y*+1, *z*; (v) −*x*, −*y*+1, −*z*; (vii) −*x*, −*y*+1, −*z*+1; (viii) −*x*+1, −*y*+1, −*z*+1; (ix) *x*+1/2, *y*+1/2, *z*; (x) *x*−1/2, *y*−1/2, *z*; (xi) *x*, *y*, *z*+1.
